# Electrospun nitrocellulose and nylon: Design and fabrication of novel high performance platforms for protein blotting applications

**DOI:** 10.1186/1754-1611-1-2

**Published:** 2007-10-10

**Authors:** Ashley E Manis, James R Bowman, Gary L Bowlin, David G Simpson

**Affiliations:** 1 Departments of Anatomy & Neurobiology, Virginia Commonwealth University, Richmond, VA 23298 USA; 2 Biomedical Engineering at Virginia Commonwealth University, Richmond, VA 23298 USA

## Abstract

**Background:**

Electrospinning is a non-mechanical processing strategy that can be used to process a variety of native and synthetic polymers into highly porous materials composed of nano-scale to micron-scale diameter fibers. By nature, electrospun materials exhibit an extensive surface area and highly interconnected pore spaces. In this study we adopted a biological engineering approach to ask how the specific unique advantages of the electrospinning process might be exploited to produce a new class of research/diagnostic tools.

**Methods:**

The electrospinning properties of nitrocellulose, charged nylon and blends of these materials are characterized.

**Results:**

Nitrocellulose electrospun from a starting concentration of < 110 mg/ml acetone deposited as 4–8 μm diameter beads; at 110 mg/ml-to-140 mg/ml starting concentrations, this polymer deposited as 100–4000 nm diameter fibers. Nylon formed fibers when electrospun from 60–140 mg/ml HFIP, fibers ranged from 120 nm-6000 nm in diameter. Electrospun nitrocellulose exhibited superior protein retention and increased sensitivity in slot blot experiments with respect to the parent nitrocellulose material. Western immunoblot experiments using fibronectin as a model protein demonstrated that electrospun nylon exhibits increased protein binding and increased dynamic range in the chemiluminescence detection of antigens than sheets of the parent starting material. Composites of electrospun nitrocellulose and electrospun nylon exhibit high protein binding activity and provide increased sensitivity for the immuno-detection of antigens.

**Conclusion:**

The flexibility afforded by electrospinning process makes it possible to tailor blotting membranes to specific applications. Electrospinning has a variety of potential applications in the clinical diagnostic field of use.

## Background

The art and technology of electrospinning has generated considerable interest in the field of tissue engineering. Studies describing various aspects and applications of the electrospinning process and patent filings for intellectual property concerning this rapidly evolving technology have undergone a remarkable expansion from 1995 to 2007. Relevant to the biological sciences and the tissue engineering fields, this technology can be used to process a variety of native [1-3] and synthetic polymers [4-6] into highly porous tissue engineering scaffolds composed of nano-scale to micron-scale diameter fibers [7], a size-scale that approaches the fiber diameters observed in the native extracellular matrix.

The physical, biochemical, and biological properties of electrospun materials can be regulated at several sites in the production process. For many polymers, physical properties, including fiber diameter, fiber alignment and pore dimension [8,9], can be regulated simply by controlling the composition of the electrospinning solvent, the air gap distance, accelerating voltage, mandrel properties and the concentration, and/or degree of chain entanglements (viscosity) present in the starting solutions [7,10]. The ability to directly regulate the physical properties of an electrospun material through the manipulation of these fundamental variables affords considerable control over the process.

The flexibility inherent to the electrospinning process makes this fabrication strategy adaptive to a variety of different fields of use. Notably, in biological applications, electrospinning shows great potential as a gateway to the development and fabrication of physiologically relevant tissue engineering scaffolds [11,12], hemostatic agents, wound care products [13], and solid phase drug and peptide delivery platforms [14]. To date, electrospinning has not penetrated to any great extent into product lines designed for diagnostic and research applications, fields of use closely allied to the more biologically applied field of tissue engineering.

Electrospun materials, by nature, exhibit an extensive surface area. The sequential deposition of the discreet, individual fibers that are formed in this process also results in a unique and complex interconnected network of pores. In this study we report that it is possible to exploit these characteristic to fabricate solid phase platforms designed for protein (*i.e*. Western blot) and/or nucleic acid detection (*i.e*. Northern blot and Southern blot). In conventional protein and nucleic acid blotting experiments, a charged sheet of nitrocellulose or nylon is used as a solid phase support [15,16]. Proteins or nucleic acids may be directly applied or transferred from a separation media, usually a polyacrylamide or agar based gel, to the solid substrate. This transfer may be affected by a vacuum, electric field or through capillary action, resulting in the binding of the protein or nucleic acid sample to the solid phase substrate. These binding events are mediated by non-specific interactions that are directly dependent upon the charge characteristics of the protein or nucleic acid of interest and the blotting platform and the surface area available for binding. Once the protein or nucleic acid of interest has been bound to the solid substrate, the sheets are blocked to reduce/eliminate non-specific binding events [17] and probed by any number of different methods to detect specific protein antigens or nucleic acid sequences [15,18-21]. These same methods can be used in conjunction with electrospinning technology to develop novel platforms for the detection of proteins and nucleic acids.

## Methods

### Viscosity measurements

All reagents obtained from Sigma Chemical Co., (St. Louis, MO, USA) unless noted. Nitrocellulose (Biorad Laboratories, Hercules, CA) was suspended and agitated for 24 hr at varying concentrations in acetone. Charged nylon (Nylon 66 derivatized with quaternary ammonium manufactured by Ambion of Austin, TX and sold as Brightstar-Plus Nylon membrane) was suspended and agitated for 24 hr in 1,1,1,3,3,3-hexaxafluoro-2-propanol (HFIP). A Brookfield RVDV-III Ultra programmable rheometer was used to measure solution viscosity.

### Electrospinning

Nitrocellulose was dissolved at various concentrations (60, 80, 100, 110, 120 and 140 mg/ml) in acetone under agitation for 24 hr. Electrospinning suspensions were loaded into a 20 ml Becton Davis syringe capped with an 18 gauge blunt tipped needle. The negative lead of a high voltage supply (Spellman CZE1000R; Spellman High Voltage Electronics Corporation) was attached by an alligator clip to the blunt tipped needle. This polarity was found to reduce drying of the acetone/nitrocellulose solution at the tip of the syringe and functioned to stabilize the Taylor cone. A 22 kV accelerating voltage was used in the electrospinning process. A Harvard perfusion pump was used to meter the delivery of the nitrocellulose solution to the electric field, the rate of delivery was set to the maximal rate that did not induce dripping from the tip of the syringe or the introduction of solvent defects in the resulting membrane (5–10 ml/hr, depending upon starting concentration).

Charged nylon was dissolved at various concentrations 60, 80, 100, 120 mg/ml in HFIP for 24 hr. Suspensions were electrospun from a 20 ml Becton Davis syringe capped with an 18 gauge blunt tipped needle. The negative lead was attached to the syringe and charged to 25 kV. The air gap distance between the source suspension and the grounded mandrel was set to 20 cm. A Harvard perfusion was used as described, the rate of solvent/polymer delivery was set at the maximal rate that did not induce dripping from the tip of the syringe (10–14 ml/hr). For all solutions, a stainless steel cylindrical mandrel (10 cm × 4.0 cm) was used as a grounded target. Each finished sheet had approximate dimensions of 15 cm × 4.0 cm.

### Scanning electron microscopy (SEM)

Membranes were sputter coated and imaged with a Zeiss EVO 50 XVP scanning electron microscope equipped with digital image acquisition. Average fiber diameter was determined from representative samples using NIH ImageTool (UTHSCSA version 3). All measurements were taken perpendicular to the long axis of electrospun fibers. Measurements were calibrated from size bars incorporated into the SEM images at the time of capture. NIH Image J software was used to conduct fiber measurements [8,9].

### Statistical evaluation

Fiber diameter data sets were screened by One-way ANOVA to determine the effects of starting conditions on fiber diameter. A Tukey test was used in the *post hoc *analysis of these data sets. Statistical significance was determined at P < 0.05.

### Slot blotting

Slot blot experiments were conducted to characterize the performance of electrospun materials. Sheets of commercially available nitrocellulose and nitrocellulose electrospun from this parent material were wet in transfer solution and mounted into a slot blot apparatus (Hoefer, San Francisco CA). Each well was rinsed 3× in transfer buffer. A series of serial dilutions of human fibronectin (Fn) was prepared in PBS and added sequentially to the blotting wells (80, 40, 20, 10, 5, 2.5, 1.25, 0.625, 0.3125, 0.1563, 0.078 μg total protein per well). After 5 minutes, the Fn solution was drawn through the blot apparatus as per slot blot manufacture's directions. The wells were each rinsed and blocked 3× with 250 μl of PBS supplemented with 1% BSA plus 0.1% Tween 20 (subsequently referred to as blocking buffer). Control lanes were incubated with blocking buffer for 5 minutes and rinsed as described.

Primary antibody (Sigma F3648) against human Fn was diluted 1:1000 in blocking buffer and applied to each lane and allowed to incubate for 5 minutes. At the conclusion of this incubation lanes were rinsed in blocking buffer 3×. Secondary antibodies (Vector Laboratories, Inc., Burlingame CA) tagged with HRP were prepared at a 1:10,000 dilution in blocking buffer and allowed to incubate for 5 minutes, the wells were re-rinsed 5× with 250 μl of blocking buffer. Samples were removed from the blot apparatus, rinsed in PBS and processed for chemiluminescence detection as per the manufacture's instructions (ECL Plus Western Blotting Detection System; Amersham, GE Health Care). Images were captured on Kodak Blue XB-1 film (Kodak). Gel images were captured with a BioRad Gel Doc 2000 system, relative optical density calculated from digital images with BioRad Gel Doc 2000 software.

### Western blot analysis

Serial dilutions of human Fn (10.0, 5.0, 2.5, 1.0, 0.50, 0.25, 0.16 0.08, 0.02 μg Fn/lane) were prepared in Laemmli buffer and separated by SDS gel electrophoresis (10% acrylamide gels, run @150 V, BioRad). Separated samples were transferred overnight (12 hr, 125 mA @4°C) onto blotting membranes. Benchmark MW Standards were used in these experiments (Invitrogen, USA).

Membranes were blocked in 5.0% non-fat milk prepared in PBS plus 0.1% Tween 20 for 30 minutes at room temperature (this formulation referred to as Western blocking buffer). Primary antibody (Sigma F3648) against human Fn was diluted 1:1000 in Western blocking buffer and applied to the membranes for 1 hr at room temperature. Membranes were rinsed 30 minutes in Western Blocking buffer under agitation using 4–5× complete changes of buffer. Secondary antibodies (Vector Laboratories, Inc., Burlingame CA) were prepared at a 1:10,000 dilution in Western blocking buffer and allowed to incubate for 1 hr at room temperature. Membranes were once again rinsed 4–5× in fresh Western blocking buffer. Samples were rinsed in PBS and then processed for chemiluminescence as described for slot blotting. Images were captured on Kodak Blue XB-1 film (Kodak).

## Results

### Electrospinning parameters: nitrocellulose

**Figure 1:**
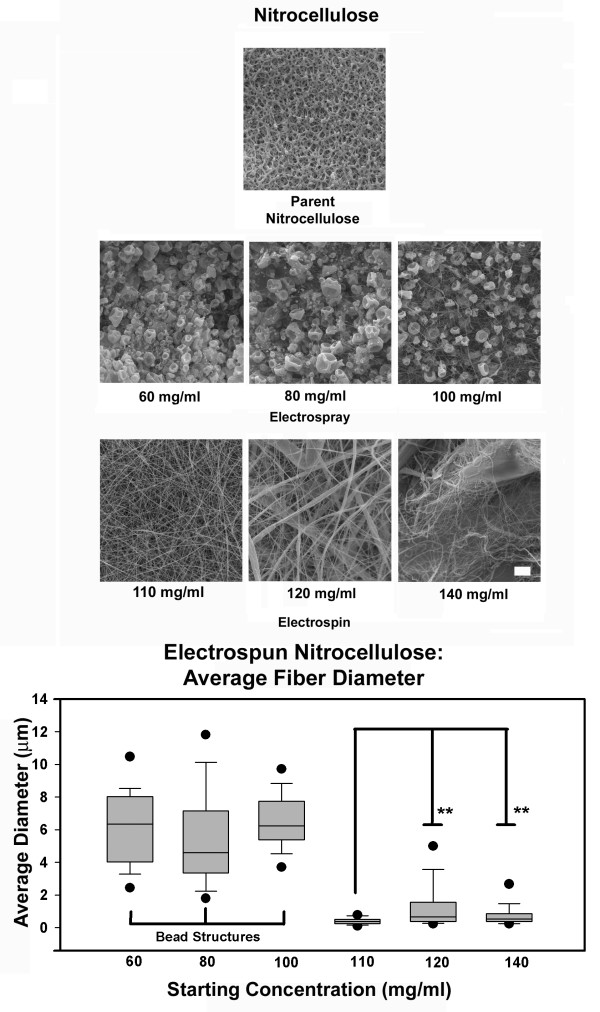
Fiber analysis: electrospun nitrocellulose. SEM images reveal that nitrocellulose forms fibers over a narrow range of electrospinning conditions. Samples electrospun from less than 110 mg/ml underwent electrospraying, a process that occurs when polymer chain entanglements are inadequate to induce fiber formation. Fibers were evident in samples prepared from 110 mg/ml, 120 mg/ml and 140 mg/ml. Note nearly uniform fiber diameters in 110 mg/ml samples, heterogeneity of diameters present in 120 mg/ml samples and solvent defects in 140 mg/ml samples (upper left of image). Fibers in 110 mg/ml solutions were smaller than fibers produced from the 120 and 140 mg/ml starting concentrations (P < 0.05). Fibers from the 120 and 140 mg solutions were not statistically different. Note the interconnected nature of the pores in samples containing fibers. Bar = 5 μm.

We examined the structural characteristics of electrospun nitrocellulose as a function of starting conditions. Samples electrospun from the 60, 80, and 100 mg/ml solutions were very similar in nature. At 60 mg/ml the bulk of the material deposited as 4–8 μm diameter beads, at 80 mg/ml foci of small diameter fibers were observed interspersed with these beads (Figure 1). The relative concentration of fibers with respect to the bead structures increased at 100 mg/ml, however, the beaded structures continued to predominate in these samples. The crenulated appearance of these beads indicates they initially form as spheres in the electrospinning (electrospray) process that contain solvent. As the solvent evaporates the beads collapse and adopt this distinctive shape [10]. At concentrations equal to or greater than 110 mg/ml the beaded structures were lost and fibers were exclusively formed in the electrospinning process (Figure 1).

Fibers electrospun from 110 mg/ml solutions were 120 nm to 1300 nm in cross sectional diameter with an average diameter of 398 nm (Figure 1). The 120 mg/ml solutions produced fibers ranging from 120 nm to 8500 nm in diameter with an average diameter of 1300 nm. The 140 mg/ml solutions produced 240 nm to 2900 nm diameter fibers with an average diameter of 725 nm. Overall, fibers in membranes prepared from the 110 mg/ml solutions were very uniform in size and, on average, were statistically smaller in diameter than fibers prepared from the 120 and 140 mg/ml solutions (P < 0.05). Solvent damage and domains of sheet-like structures (film) were evident in membranes electrospun from starting concentrations of 120 mg/ml and 140 mg/ml. We associate the appearance of these defects with upper range of electrospinning conditions that can be effectively used to produce discreet fibers. Domains that contained these defects were not included in our fiber measurements.

**Figure 2:**
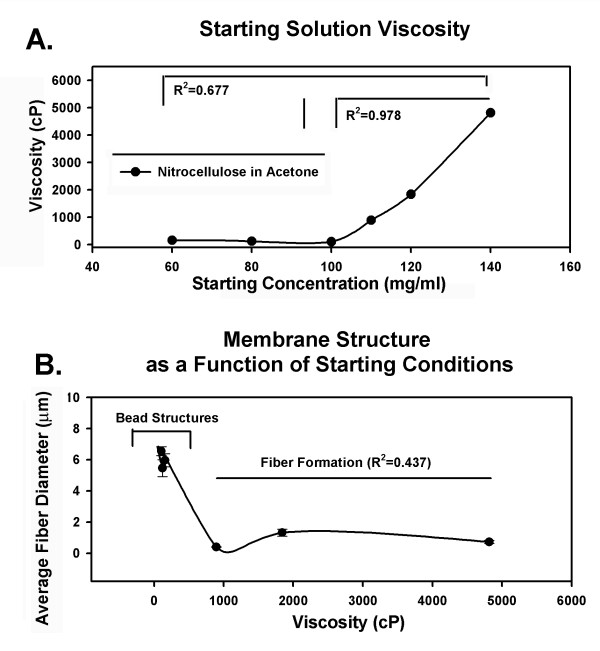
Viscosity as a function of starting concentration. (A). Viscosity was similar in solutions prepared with 60, 80 and 100 mg/ml nitrocellulose. Regression analysis of the entire data set using a 1^st ^order equation generated an R^2 ^value of 0.677, applying a 1^st ^order equation to the range of starting concentrations that produced fibers (100–140 mg/ml) generated an R^2 ^of 0.978. (B). Viscosity increased markedly from 100 to 140 mg/ml (panel A), however there was no clear relationships between fiber diameter and viscosity (panel B).

In many solvent systems average fiber diameter varies in a predictable fashion as a function of the starting polymer concentration and the viscosity of the starting solutions. However, in this system there was not a clear relationship between these parameters (Figure 2A). Not surprising, the viscosity of nitrocellulose solutions was similar at concentrations ranging from 60 mg/ml to 100 mg/ml, the conditions that produced an electrospray and membranes composed of beads. Regression analysis of the entire data set examining the relationship between starting concentration and solution viscosity using a 1^st ^order equation generated an R^2 ^value of 0.677. From 100 mg/ml to 140 mg/ml solution viscosity increased markedly. Regression analysis using a linear fit model over this limited range, essentially the conditions that resulted in fiber formation, generated an R^2 ^= 0.978 (Figure 2A). The onset of this relationship corresponded well with the onset of fiber formation in the electrospinning process. Despite this correlation, there did not appear to be a relationship between solution concentration or solution viscosity and the average fiber diameter produced during electrospinning. Regression analysis using a linear model to examine the correlation between solution concentration, over the limited range of 100–140 mg/ml, and average fiber diameter produced an R^2 ^value of 0.328 (not shown). Plotting average fiber diameter as a function of starting solution viscosity and conducting the regression analysis with a 1^st ^order equation generated an R^2 ^= 0.437 (Figure 2B).

### Slot blotting performance

**Figure 3:**
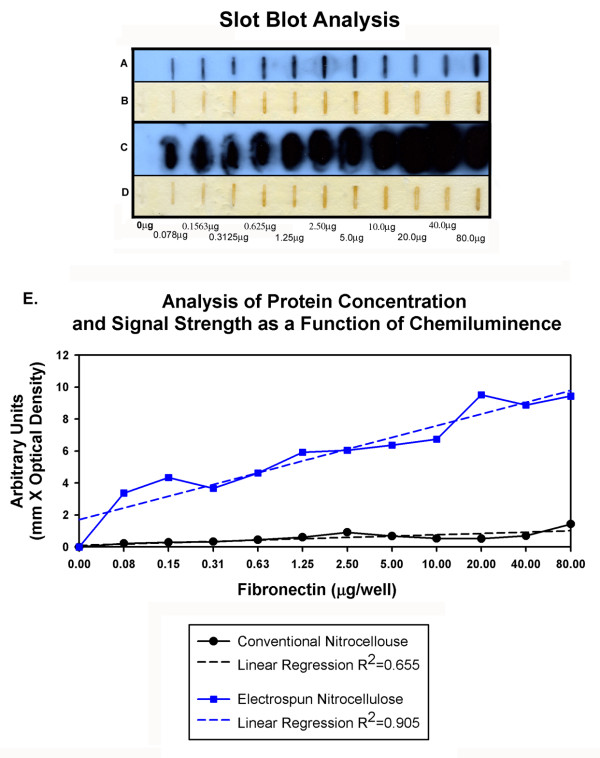
Representative Slot Blots. Chemiluminescence detection of Fn on control nitrocellulose (Lane A), corresponding image of oxidized Lumigen reaction product (B). Chemiluminescence detection of Fn on electrospun nitrocellulose (Lane C), corresponding image of oxidized Lumigen reaction product (D). Graphical illustration depicting the relative optical density present in slot blots (E). Conventional nitrocellulose blot exhibited modest increase in chemiluminescence signal as a function of increasing Fn concentration (R^2 ^= 0.655). Electrospun nitrocellulose exhibited a more pronounced signal at all protein concentrations examined. Signal increased in a nearly linear fashion over a broad range of concentrations (R^2 ^= 0.905).

To characterize the overall protein binding characteristics of electrospun nitrocellulose with respect to the parent starting material we conducted slot blot analysis. In these experiments membranes with fibers exhibiting an average cross-sectional diameter of less than 1 μm were prepared by electrospinning nitrocellulose from a starting concentration of 110 mg/ml. Serial dilutions of human Fn were then applied to the membranes and processed for detection. Staining and wash solutions were retained in the blotting wells during the incubation steps and were readily pulled through the parent and electrospun membranes when a vacuum was applied across the apparatus. Fn was detected on control nitrocellulose membranes across the sequence of concentrations tested (0.078 μg–80 μg) (Figure 3, Lanes A and B). The chemiluminescence signal associated with Fn bound to the electrospun membrane was several orders magnitude higher than the signal reported by the parent material (Figure 3, Lanes C and D). Control lanes that were treated with blocking buffer and incubated with primary and secondary antibodies did not exhibit detectable signal.

### Electrospinning parameters: charged nylon

**Figure 4:**
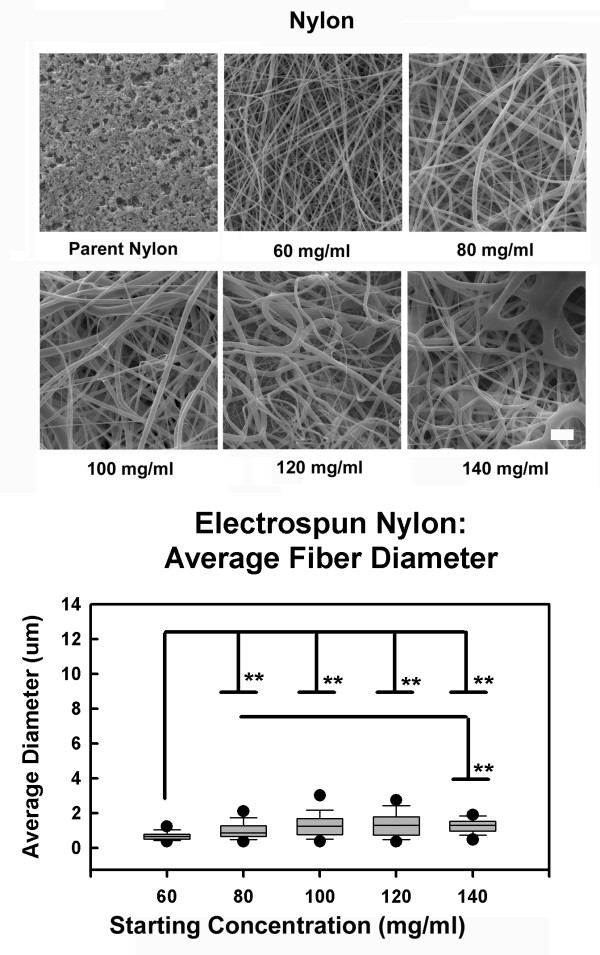
Fiber analysis: electrospun nylon. SEM images indicated that fibers were produced under all conditions assayed. Fibers electrospun from the 60 mg/ml solutions were smaller than all other treatment groups (P < 0.05) and fibers from 80 mg/ml solutions were smaller than fibers in the 140 mg/ml solutions (P < 0.05).

Charged nylon is frequently used as a solid phase substrate for protein and nucleic acid analysis. In preliminary experiments we examined the efficacy of electrospinning this material and characterized the structure of the resulting membranes. Fibers electrospun from 60 mg/ml starting suspensions were 120 nm to 1430 nm in diameter with an average diameter of 685 nm (Figure 4). At 80 mg/ml fibers were 120 nm to 3000 nm in diameter with an average of 1000 nm; at 100 mg/ml fibers were 230 nm to 6050 nm in diameter with an average of 1400 nm. The 120 mg/ml solutions produced fibers that ranged from 270 nm to 3290 nm in diameter with an average of 1400 nm; at 140 mg/ml solutions produced 370 nm to 1950 nm diameter fibers with an average of 1300 nm. Evidence of solvent induced defects and solvent welding of adjacent fibers was evident in membranes prepared from the 120 mg/ml solutions, these defects were more pronounced in the samples prepared from the 140 mg/ml solutions. Fibers produced from the 60 mg/ml solutions were smaller in diameter than all other fibers, fibers produced from the 80 mg/ml solutions were smaller than fibers produced from the 140 mg/ml solutions (Figure 4, P < 0.05).

**Figure 5:**
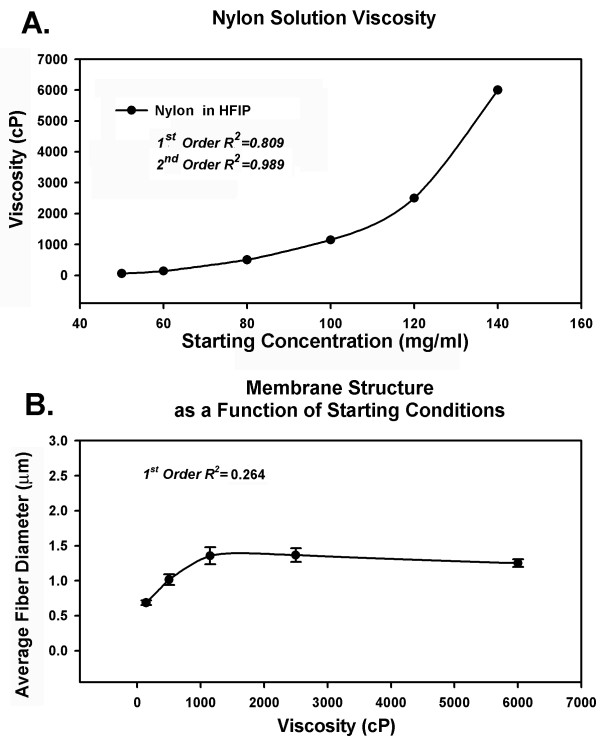
Viscosity as a function of starting concentration. (A). Solution viscosity for nylon prepared in HFIP increased as a 1^st ^order function (R^2 ^= 0.809), a 2^nd ^order equation provided an R^2 ^= 0.989. Fiber diameter did not appear to be directly related to viscosity of the starting solutions, fiber diameter remained nearly constant over a wide range of starting conditions and solution viscosities (B).

Regression analysis for viscosity as a function of starting concentration using a 1^st ^order equation generated an R^2 ^value of 0.809, a 2^nd ^order equation of these data produced an R^2 ^of 0.989 (Figure 5A). A similar analysis examining the relationships between solution viscosity and average fiber diameter produced an R^2 ^value of 0.264 for a 1^st ^order equation (Figure 5B). These data suggest that solution viscosity, but not fiber diameter, is directly related to the starting concentration of the electrospinning solutions used to process nylon.

### Western blotting

**Figure 6:**
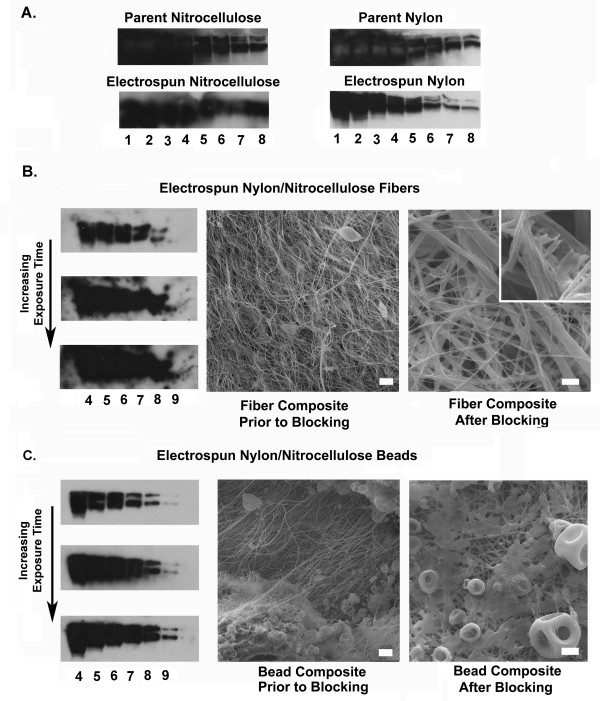
Electrospun membranes in Western blotting applications. (A). Chemiluminescence detection of Fn (lanes 1 to 9 = 10.0, 5.0, 2.5, 1.0, 0.50, 0.25, 0.16 0.08, 0.02 μg Fn/lane) on parent nitrocellulose and electrospun nitrocellulose, parent nylon and electrospun nylon (using 1 μm diameter fibers). Electrospun nitrocellulose exhibited high signal, but poor band resolution. The parent nylon material exhibited a diffuse signal in lanes loaded with the highest concentration of Fn and prominent negative images (A, lanes 1–5, top right). Contrast with electrospun nylon, this membrane exhibited sharper bands and no evidence of inverse image formation. (B) Electrospun nylon/electrospun nitrocellulose fiber composite. Note: high signal, absence of inverse white bands, but poor band resolution. We associate this result with a composite that is too thick. SEM images before (Bar = 10 μm) and after (Bar = 5 μm) blocking buffers applied to composite, inset demonstrating material coating fibers, pores between adjacent fibers remain present and open. These images suggested that electrospun nitrocellulose fibers underwent an increase in diameter during blotting (C). Electrospun nylon/electrospray nitrocellulose bead composite. This composite provided good signal detection and band resolution. SEM images before (Bar = 10 μm) and after (Bar = 5 μm) blocking buffers applied to composite.

In preliminary experiments we compared and contrasted the performance of electrospun nitrocellulose and electrospun nylon with respect to one another and the parent materials. In these conventional electroblotting experiments the loft and high surface area present in membranes composed of electrospun nitrocellulose resulted in a platform that provided high sensitivity at the expense of poor band resolution (Figure 6A). Bands were ill-defined, but intensely labeled. Large sheets of this material were difficult to handle, it was soft and tended develop folds during agitation in the staining and wash buffers.

The results of experiments conducted with electrospun nylon suggest that this material provides increased protein binding capacity and increased dynamic range with respect to the parent material in Western blotting applications (Figure 6A). Band resolution was superior to the performance of the electrospun nitrocellulose membranes. In addition, electrospun nylon was more robust and remained flat during manual manipulation and agitation. In blotting experiments the control nylon membranes developed white bands in lanes loaded with the highest concentrations of Fn. This type of inverse image can occur when A) the protein binding capacity of a blotting membrane is exceeded and/or B) the antibody dilutions are too low. This artifact was absent in the electrospun nylon membranes that were processed in parallel with the parent material (Figure 6A).

To demonstrate the utility of using electrospinning to generate unique blends of material to tailor the performance of a blotting membrane we prepared composite materials. We elected to examine 2 formulations. Electrospun nylon was prepared from a starting concentration of 60 mg/ml (average fiber diameter = 685 nm) and used as a backing material for both constructs. Next, nitrocellulose was electrospun onto the nylon backing from a starting concentration of 110 mg/ml (average fiber diameter 398 nm) or 60 mg/ml (4–8 μm diameter beads). Representative SEM images of these composites are illustrated in Figure 6. This approach allowed us to alternatively test how these two very different physical forms of nitrocellulose might perform in this application.

The nylon/nitrocellulose fiber composite exhibited high signal detection but, provided low band resolution (Figure 6B). As with pure electrospun nitrocellulose we believe the higher loft of this material contributes to the poor band resolution observed in these experiments. The electrospun nylon/electrospun nitrocellulose bead composite exhibited high sensitivity while retaining band resolution (Figure 6C). We were able to clearly detect approximately 4 fold less protein on the electrospun membrane with respect to the controls (0.02 μg Fn/lane on the electrospun composite vs. 0.08 μg Fn/lane on the parent nylon) (Figure 6C).

## Discussion

This study demonstrates the feasibility of using electrospinning to process nitrocellulose and nylon-based materials into unique membranes designed for Western (Northern and Southern) Blotting applications. We were able to generate a variety of physical states for these materials by manipulating the starting concentrations of the electrospinning solutions. For example, at low starting concentrations, nitrocellulose underwent electrospraying and deposited as 4–8 μm diameter beads, at higher starting concentrations, this polymer formed discreet sub-micron-to-micron diameter fibers. Charged nylon formed fibers over a wide range of starting concentrations, although bead formation can undoubtedly be induced by driving the initial source solution concentration below the electrospinning threshold.

### Electrospinning properties

For nitrocellulose, changes in average fiber diameter were most closely associated with the changes in the starting concentrations of the polymer used at the onset of electrospinning (Figure 1 and 2A). We believe this result may be explained by variables that are extrinsic to initial bulk solution properties. Fibers produced from the 120 and 140 mg/ml solutions exhibited a broad range of cross-sectional diameters (Figure 1). During electrospinning the charged jet produced from these concentrations was observed to episodically "extrude" several millimeters from the tip of syringe, dry and eject material in a non-uniform fashion into the electric field. This phenomenon can be expected to induce continual changes in solution viscosity within the electrospinning Taylor cone, producing fibers of varying sizes. In contrast, the charged jet produced from the 110 mg/ml solution was stable and less subject to drying at the tip of the syringe and produced more uniform fibers.

The changes in viscosity that occur as a function of nylon concentration in HFIP can be described over a wide range of conditions with a 1^st ^order equation (Figure 5A). However, our analysis suggests that fiber diameter is only directly coupled to the bulk solution properties at very low nylon concentrations (Figure 5B). At high concentrations fiber diameter was not directly correlated with solution viscosity. The variables that underlie this result remain to be defined in this system; it is possible that local changes in solution viscosity at the Taylor cone contribute to this result.

### Membrane performance

Fiber size and pore size tend to track together in the electrospinning process [7,8]. This property makes it theoretically possible to tailor membranes to specific applications. For example, for slot (dot) blotting, a membrane must exhibit high surface area and be permeable to the staining and wash solutions. Electrospun materials meet these critical characteristics. A blotting membrane composed of discreet, individual nano-to-micron diameter sized fibers has an extensive surface area [22] available for protein binding events, a physical characteristic that can be expected to increase sensitivity and the dynamic range available to this type of assay. The interconnected nature of the pores present in an electrospun membrane can be exploited to improve the penetration of proteins and the flow through of staining and wash buffers. Membranes composed of electrospun nitrocellulose exhibited superior performance in our slot blotting experiments with respect to the parent material (Figure 3). Results with membranes composed of electrospun nylon were less consistent (data not shown). We ascribe this result to the hydrophobic nature of charged nylon; it underwent drying when the vacuum was applied to blotting apparatus. In turn, this was associated with increased non-specific binding and background noise in the staining lanes, limitations that should be amenable to correction through changes in pore size and/or the development of composite materials.

### Western blotting

Membranes designed for conventional electroblotting must meet criteria similar to those described for a slot blotting membrane. As with slot blotting assays, membranes composed of electrospun nitrocellulose exhibited excellent dynamic range and sensitivity in this application. However, poor band resolution limits the utility of this composition (Figure 6A). We suspect that performance might be enhanced in this material through post-electrospinning processing with methods used in the paper industry designed to reduce wicking of materials along fibers. It may also be possible to manipulate performance through changes in fiber alignment, perhaps by creating alternating layers of arrayed (aligned) fibers [9]. Electrospun nylon exhibited sensitivity comparable to the parent membrane and exhibited distinct advantages at higher protein concentrations. As noted, the white, inverse protein bands observed in the control nylon membranes can be a consequence of excess antigen and/or the use of inadequate antibody dilutions (Figure 6A). We believe this staining artifact developed from excess protein loads present on the parent membrane. Control and electrospun membranes were processed simultaneously and exposed to the same antibody dilutions, indirect evidence that we exceeded the protein capacity of the parent membrane. The extensive surface area inherent to a fibrous construct [22] appears to increase binding capacity and clearly functions to improve the dynamic range of the assay (Figure 6B).

Electrospun nitrocellulose is a soft, flexible material that exhibits excellent signal sensitivity, but poor band resolution. Conversely, electrospun nylon withstands manual manipulation and supports high band resolution. In attempts to combine the signal sensitivity with the band resolution properties of nylon into a single membrane we tested the efficacy of two different composite materials in our Western blotting assays. Membranes composed of electrospun nylon and electrospun fibers of nitrocellulose exhibited performance limitations similar to pure preparations of electrospun nitrocellulose. The material gave excellent signal detection with poor band resolution (Figure 6B). Once again, we attribute these results to the loft of the electrospun nitrocellulose fibers. Ultimately, this limitation may be overcome by simultaneously electrospinning from separate source solutions to produce a composite membrane composed of intermingled fibers. This type of composite should exhibited less loft than the layered membrane that we tested. As alluded to earlier in this discussion, resolution may be increased through post-processing techniques designed to limit wicking/bleeding. Membranes composed of the electrospun nylon and (electrosprayed) beads of nitrocellulose provided much better performance. This material was far more compact than the fiber-fiber composition. Signal detection was approximately 4 fold better than either the parent nylon or the electrospun variant, band resolution was excellent (compare lanes 5–9 Figure 6A and 6C).

## Conclusion

Biological engineering, from a tissue-engineering prospective, can be broadly defined as a design process that seeks to capture critical features of native tissues into a template scaffold that is intended to direct the regeneration and/or reconstruction of a damaged, dysfunctional or missing organ [12]. Given this definition, we have adopted the philosophy that tissue engineering scaffolds should mimic the dimensional characteristics, tertiary structure and specific aspects of the biological activity present in the native extracellular matrix [13]. Conventional fabrication techniques typically produce biomaterials, and diagnostic tools, that are composed of structural elements that are several orders of magnitude larger than the size scale that is observed in biological systems.

The structural network of the native mammalian extracellular matrix is composed of a complex network of fibrillar protein polymers that exist on a nano-scale. Electrospinning has made it possible to fabricate a broad spectrum of materials into individual structural entities that approach this dimensional size. This new class of biomaterials exhibit unique biological [1,11], compositional [3,23] and structural properties [8,9]. The process of electrospinning exhibits a constellation of characteristics that can be exploited to regulate these fundamental variables. For example, for natural polymers like collagen [1,11] and fibrinogen [2], the electrospinning process appears to reconstitute the topological features, and cross-sectional diameters, observed in the native fibrils of these proteins. These features facilitate migration and appear to reduce the antigenic potential of these polymers [11]. The physics and chemistry of electrospinning make it possible to produce hybrid materials composed of native proteins and/or synthetic and natural protein polymers that might not otherwise co-polymerize [3,23,24]. Finally, a variety of process specific [8,9] and post-processing manipulations [25] can be implemented to regulate the biological and mechanical properties of electrospun tissue-engineering scaffolds.

In this study we adopted a biological engineering approach to ask how the specific unique advantages of the electrospinning process might be exploited to produce a new class of research/diagnostic tools. Our experiments demonstrated that electrospinning can be used to (re)engineer the physical properties and performance characteristics of nitrocellulose and charged nylon in Western blot applications. The electrospinning process imparted gross physical features that provided an extensive surface area for protein binding and a highly interconnected pore space that made these materials readily permeable to staining and wash solutions. There may be added, and entirely un-expected, advantages provided by the nano-structure of these electrospun materials. A protein bound to a fibril of electrospun nitrocellulose or electrospun nylon may adopt a very different conformation than a protein that has been immobilized to the surface of the parent starting materials.

Similar to scaffolds designed for tissue engineering, a variety of electrospinning and post-processing techniques might be applied to our system to further modulate and customize membrane performance. For example, membranes composed of polymer fibers of varying diameters and/or varying compositions can be prepared by simultaneously electrospinning from separate source solutions. In this type of construct the small diameter fibers might be used to more effectively capture small molecular weight materials, domains with larger diameter fibers can be used to capture large molecular weight materials. In more exotic applications, for example in the analysis of proteases, it may be possible to incorporate a small concentration of a specific protein, such as collagen, into an electrospun blotting platform. A sample of interest could be separated by SDS gel electrophoresis and then transferred onto the hybrid platform. Theoretically, the bound proteases would attack and degrade the incorporated protein substrate. Upon staining, much like a zymogram, the sites where active proteases were bound would appear as a clear lytic band. As an added advantage, this type of electrospun membrane could be subsequently processed for Western blot to verify enzyme identity and/or to measure enzyme content. In the clinical arena, the flexibility afforded by the electrospinning process could be exploited to produce diagnostic or research grade materials targeted to broad classes of patients, or even to specific individuals.

## List of abbreviations

BSA: Bovine Serum Albumin

Fn: Fibronectin

HFIP: 1,1,1,3,3,3-hexaxafluoro-2-propanol

Hr: hour

nm: nanometer

PBS: Phosphate Buffered Saline

SEM: scanning electron microscopy

μm: micron

## Competing interests

The authors, Manis, Bowlin, Bowman and Simpson have U.S. and International Patents Issued and Pending concerning aspects of the electrospinning process and the fabrication of blotting platforms.

## Authors' contributions

The 4 authors of this manuscript each contributed equally to the work described in this manuscript, each has read and approved the final publication. AEM, GLB and DGS conducted electrospinning experiments, JRB conducted Western and Slot blotting experiments. Image analysis and viscosity measurements by DGS and AEM.

## References

[B1] Matthews JA, Wnek GE, Simpson DG, Bowlin GL (2002). Electrospinning of collagen nanofibers. Biomacromolecules.

[B2] Wnek GE, Carr ME, Simpson DG, Bowlin GL (2003). Electrospinning of Nanofiber Fibrinogen Structures. Nano Lett.

[B3] Boland ED, Matthews JA, Pawlowski KJ, Simpson DG, Wnek GE, Bowlin GL (2004). Electrospinning collagen and elastin: preliminary vascular tissue engineering. Front Biosci.

[B4] Boland ED, Coleman BD, Barnes CP, Simpson DG, Wnek GE, Bowlin GL (2005). Electrospinning polydioxanone for biomedical applications. Acta Biomater.

[B5] Boland ED, Wnek GE, Simpson DG, Pawlowski KJ, Bowlin GL (2001). Tailoring Tissue Engineering Scaffolds Using Electrostatic Processing Techniques: A Study of Poly(Glycolic Acid). J Macromol Sci.

[B6] Yang F, Murugan R, Wang S, Ramakrishna S (2005). Electrospinning of nano/micro scale poly(L-lactic acid) aligned fibers and their potential in neural tissue engineering. Biomaterials.

[B7] Doshi J, Reneker DH (1995). Electrospinning process and applications of electrospun fibers. J Electrostat.

[B8] Ayres CE, Bowlin GL, Henderson SC, Taylor L, Schultz J, Alexander JK, Telemeco TA, Simpson DG (2006). Modulation of Anisotropy in Electrospun Tissue Engineering Scaffolds: Analysis of Fiber Alignment by the Fast Fourier Transform. Biomaterials.

[B9] Ayres CE, Bowlin GL, Pizinger R, Taylor LT, Keen KA, Simpson DG (2007). Incremental Changes in Anisotropy: Modulation of Material Properties in Electrospun Tissue Engineering Scaffolds. Acta Biomater.

[B10] Deitzel JM, Kleinmeyer J, Harris D, Tan NCB (2001). The effect of processing variables on the morphology of electrospun nanofibers and textiles. Polymer.

[B11] Telemeco TA, Ayres CE, Bowlin GL, Wnek G, Boland G, Cohen N, Baumgarten CM, Mathews J, Simpson DG (2005). Regulation of Cellular Infiltration into Tissue Engineering Scaffolds Composed of Submicron Diameter Fibrils Produced by Electrospinning. Acta Biomater.

[B12] Simpson DG, Bowlin GL (2006). Tissue-engineering scaffolds: can we re-engineer mother nature?. Expert Rev Med Devices.

[B13] Simpson DG (2006). Dermal templates and the wound-healing paradigm: the promise of tissue regeneration. Expert Rev Med Devices.

[B14] Kenawy E, Mansfield K, Bowlin GL, Simpson DG, Wnek GE (2002). New Drug Delivery System: Control Release of Tetracycline Hydrochloride as a Model Drug from Electrospun Fibers of Poly(lactic acid) and Poly(ethylene vinyl acetate). J Control Release.

[B15] Towbin H, Staehelin T, Gordon J (1979). Electrophoretic transfer of proteins from polyacrylamide gels to nitrocellulose sheets: procedure and some applications. Proc Natl Acad Sci USA.

[B16] Elkon KB, Chu JL (1984). Counter immunoblotting: detection of non-denatured or denatured antigens in antibody-containing agarose gels following polyacrylamide gel electrophoresis. J Immunol Methods.

[B17] Batteiger B, Newhall WJ, Jones RB (1982). The use of Tween 20 as a blocking agent in the immunological detection of proteins transferred to nitrocellulose membranes. J Immunol Methods.

[B18] De Blas AL, Cherwinski HM (1983). Detection of antigens on nitrocellulose paper immunoblots with monoclonal antibodies. Anal Biochem.

[B19] Judd RC (1987). Radioiodination and 125I-labeled peptide mapping of proteins on nitrocellulose membranes. Anal Biochem.

[B20] O'Connor CG, Ashman LK (1982). Application of the nitrocellulose transfer technique and alkaline phosphatase conjugated anti-immunoglobulin for determination of the specificity of monoclonal antibodies to protein mixtures. J Immunol Methods.

[B21] Turner BM (1983). The use of alkaline-phosphatase-conjugated second antibody for the visualization of electrophoretically separated proteins recognized by monoclonal antibodies. J Immunol Methods.

[B22] Frenot A, Chronakis IS (2003). Polymer nanofibers assembled by electrospinning. Current Opinion In Colloid & Interface Science.

[B23] Smith MJ, McClure MJ, Sell SA, Barnes CP, Walpoth BH, Simpson DG, Bowlin GL (2007). Suture-reinforced electrospun polydioxanone – elastin small-diameter tubes for use in vascular tissue engineering: a feasibility study. Acta Biomater.

[B24] Sell SA, McClure MJ, Barnes CP, Knapp DC, Walpoth BH, Simpson DG, Bowlin GL (2006). Electrospun polydioxanone-elastin blends: potential for bioresorbable vascular grafts. Biomed Mater.

[B25] Barnes CP, Pemble CW, Brand DD, Simpson DG, Bowlin GL (2007). Cross-linking Electrospun Type II Collagen Tissue Engineering Scaffolds with Carbodiimide in Ethanol. Tissue Eng.

